# Comparative Cytogenetics and Neo-Y Formation in Small-Sized Fish Species of the Genus Pyrrhulina (Characiformes, Lebiasinidae)

**DOI:** 10.3389/fgene.2019.00678

**Published:** 2019-08-02

**Authors:** Renata Luiza Rosa de Moraes, Alexandr Sember, Luiz Antônio Carlos Bertollo, Ezequiel Aguiar de Oliveira, Petr Ráb, Terumi Hatanaka, Manoela Maria Ferreira Marinho, Thomas Liehr, Ahmed B. H. Al-Rikabi, Eliana Feldberg, Patrik F. Viana, Marcelo de Bello Cioffi

**Affiliations:** ^1^Laboratório de Citogenética de Peixes, Departamento de Genética e Evolução, Universidade Federal de São Carlos (UFSCar), São Carlos, Brazil; ^2^Laboratory of Fish Genetics, Institute of Animal Physiology and Genetics, Czech Academy of Sciences, Liběchov, Czechia; ^3^Secretaria de Estado de Educação de Mato Grosso – SEDUC-MT, Cuiabá, Brazil; ^4^Museu de Zoologia da Universidade de São Paulo, (MZUSP), São Paulo, Brazil; ^5^Institute of Human Genetics, University Hospital Jena, Jena, Germany; ^6^Laboratório de Genética Animal, Instituto Nacional de Pesquisas da Amazônia, Coordenação de Biodiversidade, Manaus, Brazil

**Keywords:** fishes, molecular cytogenetics, sex chromosome, chromosomal painting, comparative genomic hybridization (CGH), karyotype evolution

## Abstract

Although fishes have traditionally been the subject of comparative evolutionary studies, few reports have concentrated on the application of multipronged modern molecular cytogenetic techniques (such as comparative genomic hybridization = CGH and whole chromosome painting = WCP) to analyze deeper the karyotype evolution of specific groups, especially the historically neglected small-sized ones. Representatives of the family Lebiasinidae (Characiformes) are a notable example, where only a few cytogenetic investigations have been conducted thus far. Here, we aim to elucidate the evolutionary processes behind the karyotype differentiation of *Pyrrhulina* species on a finer-scale cytogenetic level. To achieve this, we applied C-banding, repetitive DNA mapping, CGH and WCP in *Pyrrhulina semifasciata* and *P. brevis*. Our results showed 2n = 42 in both sexes of *P. brevis*, while the difference in 2n between male and female in *P. semifasciata* (♂41/♀42) stands out due to the presence of a multiple X_1_X_2_Y sex chromosome system, until now undetected in this family. As a remarkable common feature, multiple 18S and 5S rDNA sites are present, with an occasional synteny or tandem-repeat amplification. Male-*vs*.-female CGH experiments in *P. semifasciata* highlighted the accumulation of male-enriched repetitive sequences in the pericentromeric region of the Y chromosome. Inter-specific CGH experiments evidenced a divergence between both species’ genomes based on the presence of several species-specific signals, highlighting their inner genomic diversity. WCP with the *P*. *semifasciata*-derived Y (PSEMI-Y) probe painted not only the entire metacentric Y chromosome in males but also the X_1_ and X_2_ chromosomes in both male and female chromosomes of *P. semifasciata.* In the cross-species experiments, the PSEMI-Y probe painted four acrocentric chromosomes in both males and females of the other tested *Pyrrhulina* species. In summary, our results show that both intra- and interchromosomal rearrangements together with the dynamics of repetitive DNA significantly contributed to the karyotype divergence among *Pyrrhulina* species, possibly promoted by specific populational and ecological traits and accompanied in one species by the origin of neo-sex chromosomes. The present results suggest how particular evolutionary scenarios found in fish species can help to clarify several issues related to genome organization and the karyotype evolution of vertebrates in general.

## Introduction

South American miniature freshwater fishes cover, by definition, the species that do not exceed 26 mm in the standard length, yet most of them reach the maturity with a length of 20 mm (Weitzman and Vari, 1988). Such a small size limited or hampered especially cytogenetic investigations in these fishes over years. It is also the case of the Lebiasinidae family (Characiformes), whose representatives generally range from 16 to 70 mm in length. They are mainly distributed in the isolated streams of Central America (Panama and Costa Rica) and in almost all South American countries, except for Chile (Weitzman and Weitzman, 2003). Lebiasinidae branches to two subfamilies: Lebiasininae and Pyrrhulininae, which comprise seven genera and 77 recognized species (Froese and Pauly, 2018). Lebiasininae is formed by three genera: *Lebiasina* (18 recognized species), *Piabucina* (nine species), and a monotypic *Derhamia* (Weitzman and Weitzman, 2003; Froese and Pauly, 2018). Pyrrhulininae is considerably more diverse group on the species level (Netto-Ferreira and Marinho, 2013), encompassing four genera: *Nannostomus* (20 species), *Pyrrhulina* (18 species), *Copella* (6 species), and *Copeina* (2 species) (Weitzman and Weitzman, 2003; Froese and Pauly, 2018). Fishes from this subfamily experienced gradual decrease in the body size during their evolution, resulting in many miniaturized taxa (Netto-Ferreira and Marinho, 2013).

Lebiasinidae was formerly considered to be phylogenetically related to Erythrinidae, Ctenoluciidae, and Hepsetidae, due to sharing of some particular morphological similarities (Buckup, 1998). Nonetheless, more recent robust molecular phylogenetic analyses indicated that Erythrinidae and Hepsetidae are in fact not closely related to Lebiasinidae; instead, the close relationship between Lebiasinidae and Ctenoluciidae was demonstrated (Arcila et al., 2017; Arcila et al., 2018). However, while providing significant advances to this issue, these relationships still require complementary studies for the comprehensive understanding of the evolutionary history among its evolutionary lineages. In this context, conventional and molecular cytogenetic studies have brought valuable contributions to clarify the evolutionary relationships among phylogenetically related fish lineages (reviewed in Cioffi et al., 2018). However, like many other Neotropical fish groups with many representatives of small to miniature body size, the Lebiasinidae family was subject of only limited cytogenetic effort conducted thus far. The very small size of its species, especially the Pyrrhulininae ones, pose a significant challenge as it is notoriously difficult to obtain satisfactory chromosomal preparations, and therefore, most of the available data are limited only to the description of the haploid and/or diploid chromosome numbers (n/2n) in some species, with particularly 2n ranging from 22 in *N. unifasciatus* to 46 in *N. trifasciatus* (Scheel, 1973; Oliveira et al., 1991; Arai, 2011). However, a recent study employing the combined conventional and molecular cytogenetic approach in the two *Pyrrhulina* species (*P. australis* and *Pyrrhulina* aff. *australis*) has been conducted. Despite the fact that both species have been found to share the same 2n (40), without any karyotype differentiation between the sexes, interspecific CGH experiments were convincing enough to demonstrate some degree of genomic divergence, as inferred from a range of non-overlapping species-specific signals (Moraes et al., 2017).

In recent years, modern molecular cytogenetic techniques including whole chromosome painting (WCP) and comparative genomic hybridization (CGH) have been effective in broadening our understanding of the genome evolution and organization in fishes, allowing us to gain more detailed insights into a number of evolutionary issues. Specifically, both techniques have been used for the investigation of genomic divergence among related species (Nagamachi et al., 2010; Symonová et al., 2013; Moraes et al., 2017; Sember et al., 2018b) and to track the origin and evolution of sex chromosomes (Phillips et al., 2001; Henning et al., 2011; Cioffi et al., 2013; Freitas et al., 2018; Oliveira et al., 2018).

The present work aims to extend our understanding of the chromosomal evolutionary processes within the Lebiasinidae family, particularly by a deep investigation of the evolutionary relationships within the *Pyrrhulina* genus. For this, we applied C-banding, repetitive DNA mapping, CGH, and WCP in two species of *Pyrrhulina* – *P. semifasciata* and *P. brevis*. Our results strongly indicated the presence of a multiple X_1_X_2_Y sex chromosome system in *P. semifasciata*, which clearly emerged from a relatively recent centric fusion event with the signs of emerging male-specific region around the fusion point. In addition, the data obtained also highlight the high chromosomal dynamics within the investigated *Pyrrhulina* species, probably driven by the small population sizes and/or certain ecological properties of these small fishes.

## Materials and Methods

### Animals

**Figure 1:**
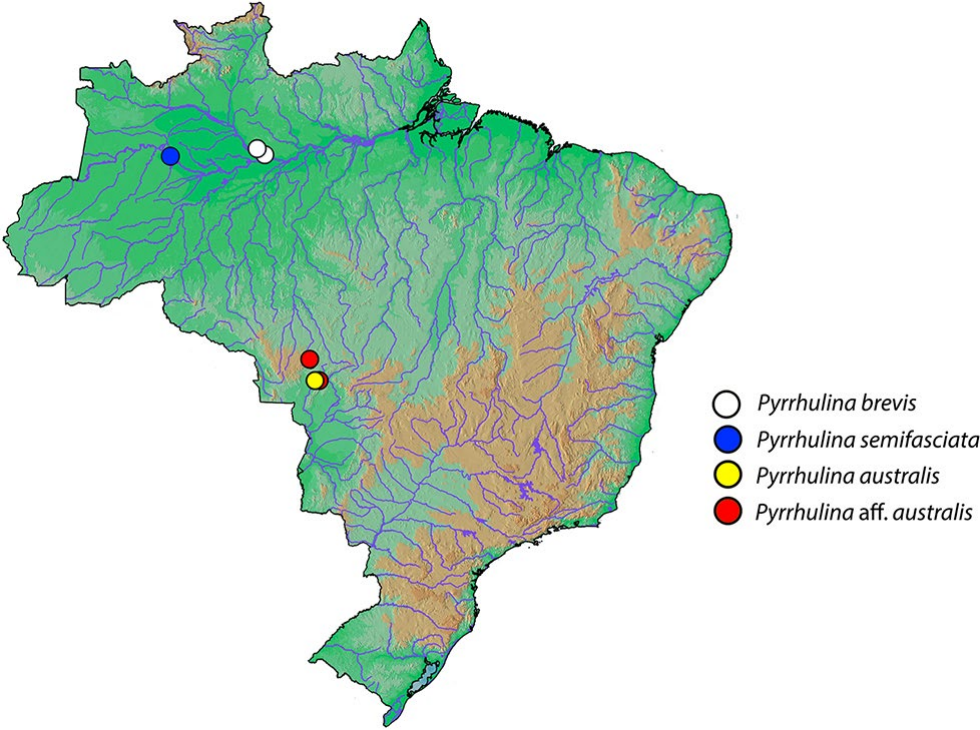
Brazilian map showing the collection sites of *Pyrrhulina brevis* (white circle), *Pyrrhulina semifasciata* (blue circles), *Pyrrhulina australis* (yellow circle), and *Pyrrhulina* aff. *australis* (red circles). The last two species were cytogenetically investigated in Moraes et al. (2017).

The number and sex of individuals investigated, collection sites, and the respective deposit numbers are presented in **Figure 1** and **Table 1**. The individuals were collected with the authorization of the Brazilian environmental agency ICMBIO/SISBIO (license no. 48628-2) and SISGEN (A96FF09). All species were properly identified by morphological criteria, and specimens were deposited in the fish collections of the Museu de Zoologia da Universidade de São Paulo (MZUSP) under the voucher numbers (119077, 119079, 123073, 123077, and 123080). The experiments followed ethical and anesthesia conducts and were approved by the Ethics Committee on Animal Experimentation of the Universidade Federal de São Carlos (process number CEUA 1853260315).

**Table 1:** Brazilian collection sites of the *Pyrrhulina* species analyzed, with the sample sizes (*N*).

Species	Collection site	N
*Pyrrhulina australis*	- Branco river (MT) – Paraguai river Basin	(30♀; 18 ♂)
*Pyrrhulina* aff. *australis*	- St. Antônio stream (MT) – Amazon river Basin	(22 ♀; 16 ♂)
*Pyrrhulina* aff. *australis*	- Branco river (MT) – Paraguai river Basin	(09 ♀; 20 ♂)
*Pyrrhulina brevis*	- Adolfo Ducke Rerserve- Igarapé from Barro Branco	(13 ♀; 17 ♂)
*Pyrrhulina semifasciata*	- Tefé River (AM) – Amazon river Basin	(07 ♀; 12 ♂)

### Chromosome Preparation and Analysis of Constitutive Heterochromatin

Mitotic chromosomes were obtained from kidney cells by the protocol described in Bertollo et al. (2015). Visualization of the amount and distribution of constitutive heterochromatin was done by C-banding according to Sumner (1972).

### Preparation of FISH Probes Derived From Repetitive Sequences

The 5S rDNA probe included 120 base pairs (bp) of the 5S rDNA gene coding region and the 200 bp long non-transcribed spacer (NTS) (Pendás et al., 1994). The 18S rDNA probe corresponded to a 1,400-bp-long segment of the 18S rDNA coding region (Cioffi et al., 2009). The 18S and 5S rDNA probes were directly labeled with the Nick-Translation Mix (Roche, Mannheim, Germany) – 18S rDNA with Spectrum Green-dUTP and 5S rDNA with Spectrum Orange-dUTP (both Vysis, Downers Grove, USA), according to the manufacturer’s instructions. (CA)_15_ and (GA)_15_ microsatellite probes were directly labeled with Cy3 during the synthesis according to Kubát et al. (2008).

### Fluorescence *In Situ* Hybridization (FISH) for Repetitive DNA Mapping

Fluorescence *in situ* hybridization (FISH) was performed under high-stringency conditions as described in Yano et al. (2017a). Briefly, the chromosome preparations were incubated with RNase A (40 μg/ml) for 1.5 h at 37°C. After denaturation of the chromosomal DNA in 70% formamide/2x SSC at 70°C, slides were dehydrated in an ethanol series (70, 85 and 100%), 2 min each. 20 µl of the hybridization mixture (100 ng of each probe, 50% deionized formamide and 10% dextran sulfate) were dropped on the slides, and the hybridization was performed for 14 h at 37°C in a moist chamber containing 2x SSC (pH = 7.0). The post-hybridization wash was carried out with 1x SSC for 5 min at 42°C. Finally, the chromosomes were counterstained with DAPI (1.2 µg/ml) and mounted in antifade solution (Vector, Burlingame, CA, USA).

### Preparation of Probes for Comparative Genomic Hybridization (CGH)

The gDNAs of males and females of *P. semifasciata* and *P. brevis* were extracted from liver tissue by a standard phenol-chloroform-isoamyl alcohol method (Sambrook and Russell, 2001). Two different experimental designs were used for this study. In the first set of experiments, we focused on intraspecific comparisons. In this case, male and female gDNAs of *P. semifasciata* and *P. brevis* were labeled and hybridized against the chromosomal background of males from *P. semifasciata* and *P. brevis,* respectively. Male gDNAs were labeled with digoxigenin-11-dUTP using DIG-Nick Translation Mix (Roche, Mannheim, Germany), while female gDNAs were labeled with biotin-16-dUTP using BIO-Nick Translation Mix (Roche, Mannheim, Germany). The final hybridization mixture for each slide contained 500 ng of each male- and female-derived labeled gDNA and 25 μg of unlabeled female-derived C_0_t-1 DNA (to block the shared repetitive sequences; prepared according to Zwick et al., 1997), dissolved in 20 μl of the hybridization buffer (50% formamide, 2× SSC, 10% SDS, 10% dextran sulfate and Denhardt´s buffer, pH 7.0). In the second set of experiments, we focused on the interspecific genomic comparisons; hence, we co-hybridized 500 ng of male-derived gDNA of *P. semifasciata* (labeled with digoxigenin-11-dUTP) with 500 ng of male-derived gDNA of *P. brevis* (labeled with biotin-16-dUTP) on the chromosomal background of both species. In this case, the final probe cocktail for each slide contained also 15 μg of female-derived C_0_t-1 DNA from *P. semifasciata* and 15 μg of female-derived C_0_t-1 DNA from *P. brevis*.

### FISH Used for CGH

CGH experiments were performed according to Symonová et al. (2015). Briefly, the slides were aged for 1–2 h at 60°C, followed by a treatment with RNase A (200 µg/ml; 90 min at 37°C in a wet chamber) and with pepsin (50 µg/ml; 3 min at 37°C). Chromosomes were denatured in 75% formamide in 2xSSC at 74°C for 3 min, while the probes were denatured at 86°C for 6 min, chilled on ice (10 min) and then applied on the slides. Hybridization was done for 3 days in a humid chamber (37°C). Subsequently, non-specific hybridization was removed by a stringent washing at 44°C, twice in 50% formamide/2xSSC (10 min each) and three times in 1xSSC (7 min each), and then rinsed in 2xSSC at room temperature. The hybridization signals were detected with Anti-Digoxigenin-Rhodamin (Roche, Mannheim, Germany) diluted in 0.5% bovine serum albumin (BSA) in PBS, and avidin-FITC (Sigma, St. Louis, MO, USA) diluted in PBS containing 10% normal goat serum (NGS). Four final washes were performed at 44°C in 4xSSC/0.1% Tween, 7 min each. Finally, the chromosomes were counterstained with DAPI (1.2 µg/ml) and mounted in an antifade solution (Vector, Burlingame, CA, USA).

### Chromosome Microdissection, Probe Preparation, and Labeling

Twenty copies of the Y chromosome from *P. semifasciata* (hereafter designated as PSEMI-Y) were manually microdissected using the glass needles, under an inverted microscope (Zeiss Axiovert 135). The chromosomes were amplified by degenerate oligonucleotide primed-PCR (DOP-PCR), following the protocol described in Yang et al. (2009). Then, 1 μl of the primary amplification product was used as a template DNA for a secondary labeling DOP-PCR with Spectrum Orange-dUTP (Vysis, Downers Grove, USA) in 30 cycles, following Yang and Graphodatsky (2009). The final probe mixture for one slide contained 500 ng of the PSEMI-Y probe and 30µg of C_0_t-1 DNA isolated from *P. semifasciata* female genome.

### FISH Used for Whole Chromosome Painting

Chromosomal preparations of males and females of *P. semifasciata, P. brevis,* and two other *Pyrrhulina* species (*P. australis* and *P.yrrhulina* aff. *australis*) were used for Zoo-FISH experiments with the PSEMI-Y probe. The hybridization procedures followed Yano et al. (2017a). Hybridization was performed for 48 h at 37°C in a moist chamber. The post-hybridization wash was carried out with 1xSSC for 5 min at 65°C, and in 4xSSC/Tween (RT), and the chromosomes were mounted with DAPI (1.2 µg/ml) in antifade as described above.

### Microscopy and Image Processing

At least 30 metaphase spreads per individual were analyzed to confirm the 2n, karyotype structure and the FISH results. Images were captured using an Olympus BX50 epifluorescence microscope (Olympus Corporation, Ishikawa, Japan) with the CoolSNAP system software and the images were processed using Image Pro Plus 4.1 Software (Media Cybernetics, Silver Spring, MD, USA). Final images were optimized and arranged using Adobe Photoshop, version 7.0. Chromosomes were classified as metacentric (m), submetacentric (sm), subtelocentric (st), or acrocentric (a), according to their arm ratios (Levan et al., 1964).

## Results

### Karyotype Analysis and Heterochromatin Distribution

**Figure 2:**
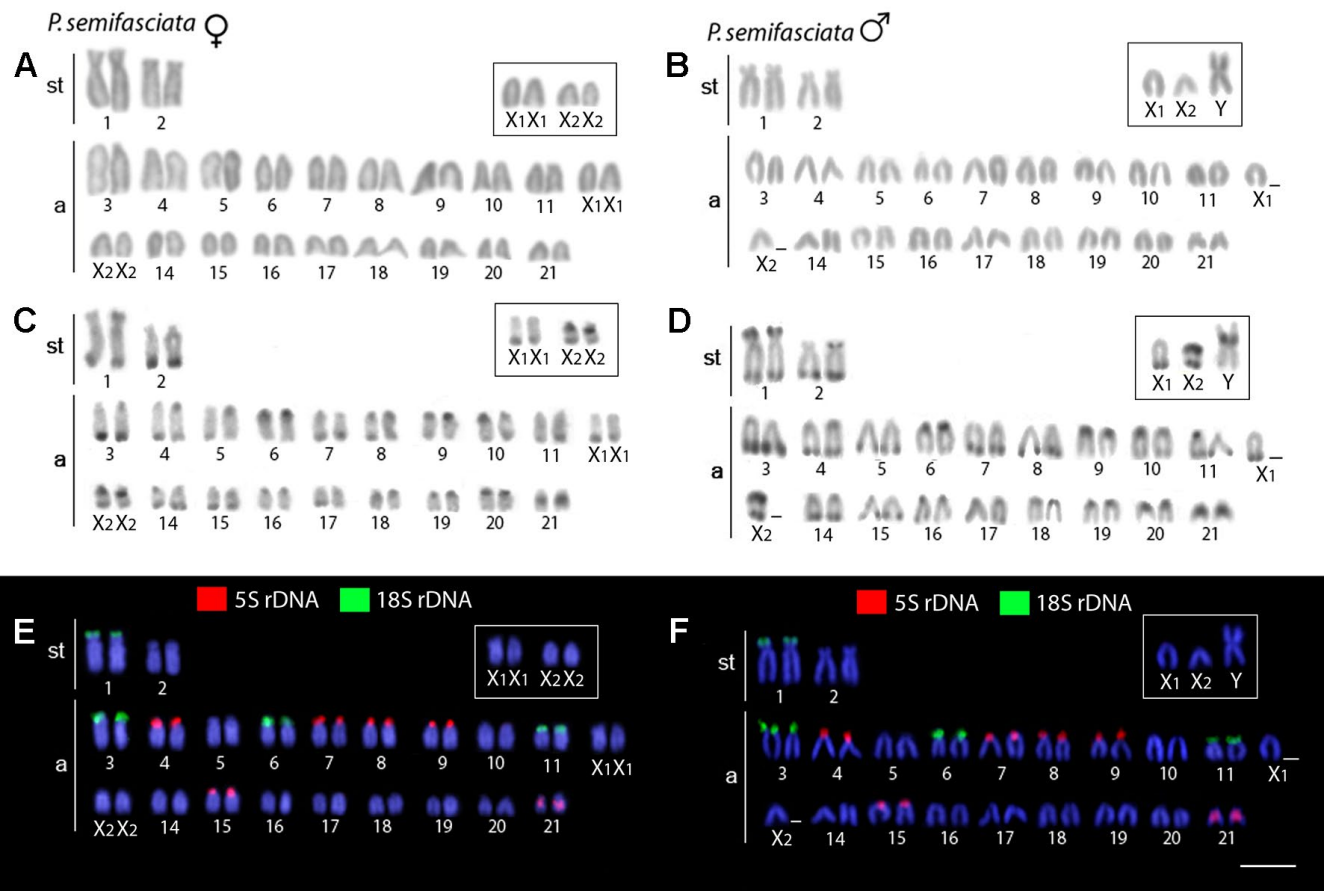
Karyotypes of *Pyrrhulina semifasciata* (female and male) arranged from chromosomes after different cytogenetic procedures. Giemsa staining in female **(A)** and male **(B)**, C-banding in female **(C)** and male **(D)**, dual-color FISH with 18S (green) and 5S (red) rDNA probes in female **(E)** and male **(F)**. Chromosomes are counterstained with DAPI (blue). Bar = 5 µm.

**Figure 3:**
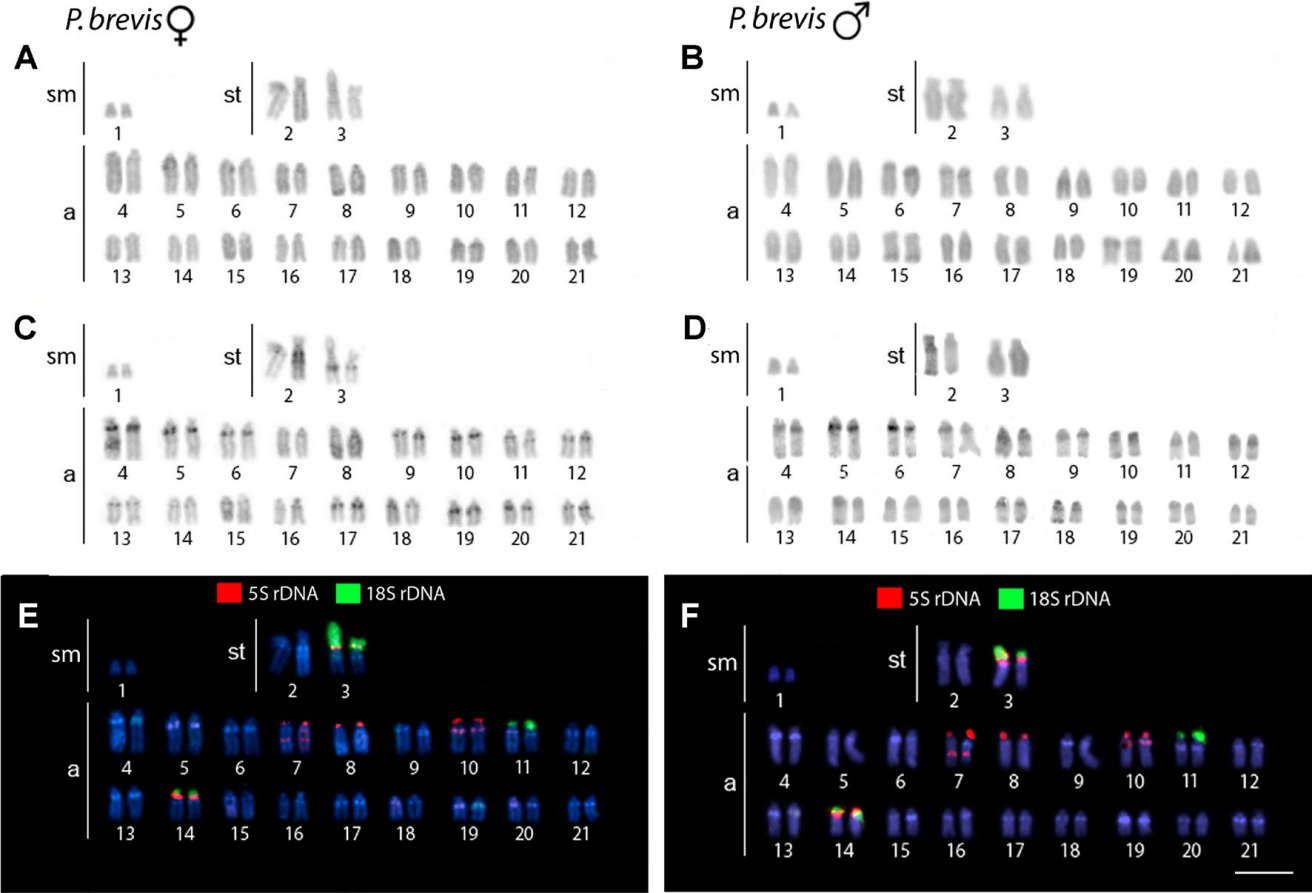
Karyotypes of *Pyrrhulina brevis* (female and male) arranged from chromosomes after different cytogenetic protocols. Giemsa staining in female **(A)** and male **(B)**, C-banding in female **(C)** and male **(D)**, dual-color FISH with 18S (green) and 5S (red) rDNA probes in female **(E)** and male **(F)**. Chromosomes are counterstained with DAPI (blue). Bar = 5 µm.

The karyotype of *P. semifasciata* was composed of 2n = 41, 1m + 4st + 36a in males, and 2n = 42, 4st + 38a in females (**Figure 2**). However, all *P. brevis* individuals displayed 2n = 42 and the karyotype composed of 2sm + 4st + 36a, both in males and females (**Figure 3**). The distribution of constitutive heterochromatin was restricted to the centromeric and telomeric regions of several chromosomes in both species, but the intensity of C-bands was more pronounced in *P. semifasciata*. *P. brevis* further displayed conspicuous interstitial C-bands, which were absent in *P. semifasciata* (**Figures 2** and **3**).

### Chromosomal Mapping of Repetitive DNA Markers

**Figure 4:**
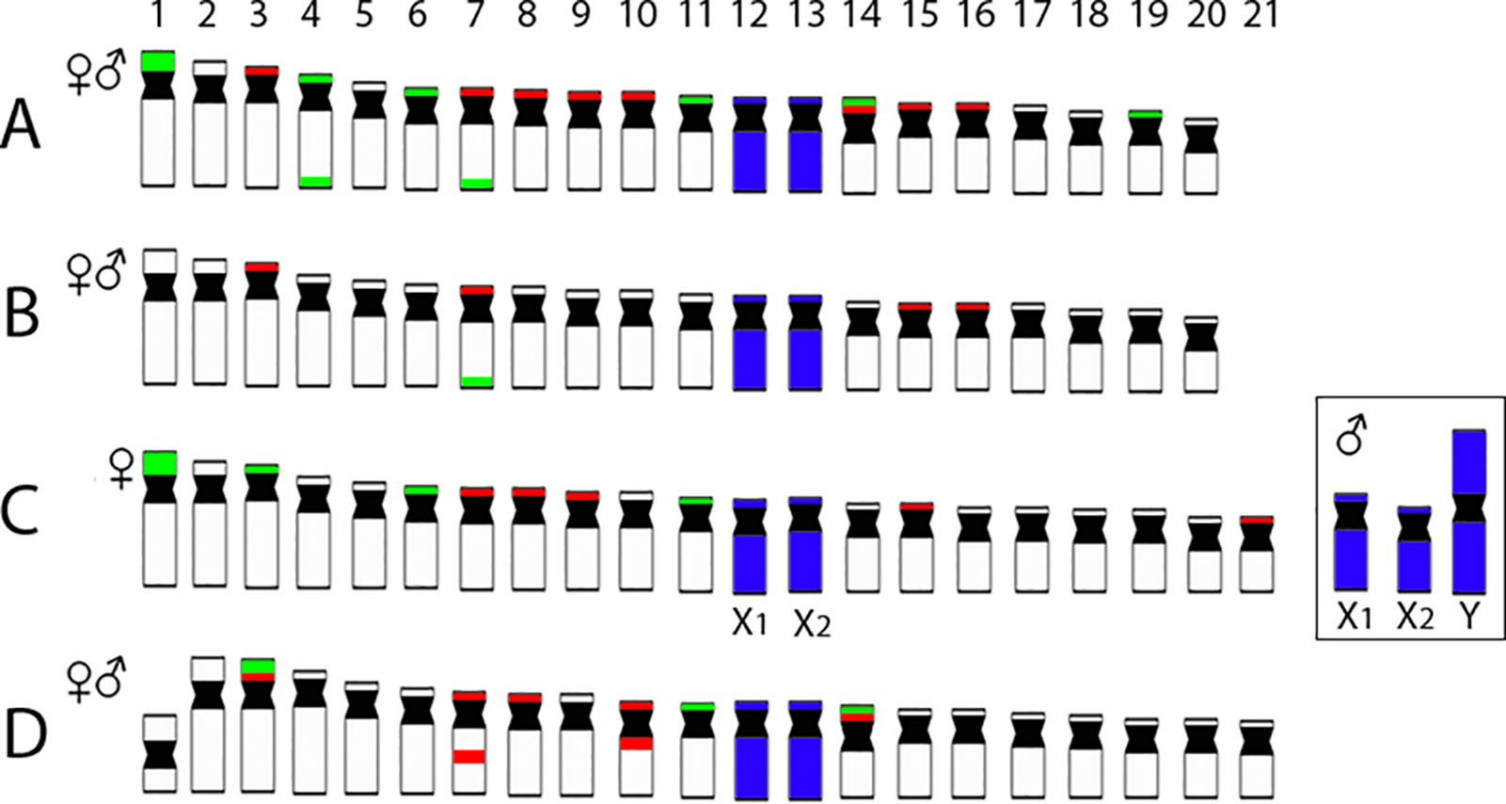
Representative idiograms of *Pyrrhulina* species showing the distribution of 18S (green) and 5S rDNA (red) sites on the chromosomes of *P. australis* **(A)**, *Pyrrhulina* aff. *australis* **(B)** (based on our previous study; Moraes et al. (2017), and *P. semifasciata* **(C)** and *P. brevis* **(D)** (this study). Dark blue indicates the chromosomes painted with the PSEMI-Y probe. Bar = 5 µm.

Dual-color FISH with 5S and 18S rDNA probes revealed that the investigated species differ notably by their patterns of distribution for both multigene families, yet they share a presence of multiple sites for both ribosomal clusters. In *P. semifasciata*, the 18S rDNA cistrons were found to cover short (*p*) arms of the largest (st) chromosome pair in the karyotype as well as the *p*-arms of three acrocentric pairs (nos. 3, 6, and 11), while 5S rDNA signals occupied the *p*-arms of five acrocentric pairs (nos. 4, 7, 8, 9, and 15), with yet another sixth acrocentric pair (no. 21) bearing an interstitial 5S cluster (**Figures 2** and **4**). In contrast to *P. semifasciata*, where none of the rDNA signals occured in synteny or even in the adjacent regions, two out of three pairs of 18S-bearing chromosomes in *P. brevis* (st pair no. 3 and a pairs nos. 11 and 14) bore also an adjacent 5S rDNA site on their *p*-arms (pairs nos. 3 and 14). At the same time, pair no. 3 exhibited a remarkable 18S rDNA site amplification accompanied by an extensive size heteromorphism between homologs (**Figures 3** and **4**). Besides chromosome pairs nos. 3 and 14, there were another three acrocentric pairs bearing 5S rDNA tandem repeats (pairs nos. 7, 8, and 10). Interestingly, pairs 7 and 10 encompassed double 5S rDNA sites—one occupying *p*-arms and the second being placed interstitially on the long (*q*) arms on both chromosome pairs (**Figures 3** and **4**).

**Figure 5:**
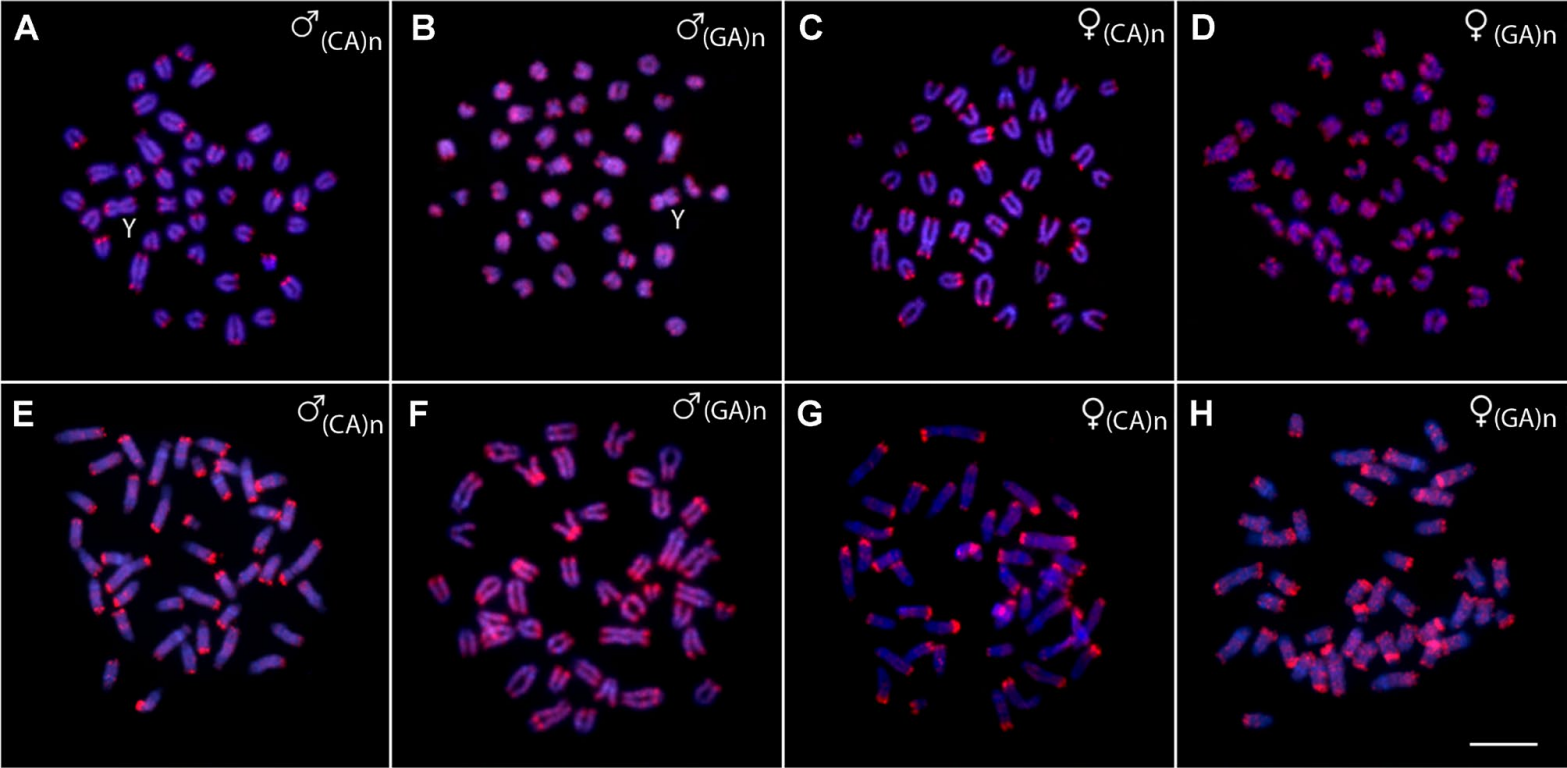
Metaphases from males and females of *Pyrrhulina semifasciata* **(A**-**D)** and *Pyrrhulina brevis* **(E**-**H)** hybridized with the microsatellite probes (CA)_15_ and (GA)_15_, showing the general distribution pattern of these repetitive DNAs on the chromosomes. Bar = 5 µm.

The chromosomal mapping of the microsatellite motif (CA)_15_ showed a prominent clustering in the telomeric sites of all chromosomes, especially on *q*-arms, while few distinct interstitial accumulations were also apparent, especially in *P. brevis*. On the other hand, (GA)_15_ motif displayed more scattered distribution along the chromosome complement of both species, though a strong preference for telomeric regions can be also inferred for this motif (**Figure 5**).

### Detection of the Male-Specific Region and Interspecific Genomic Divergence by CGH

**Figure 6:**
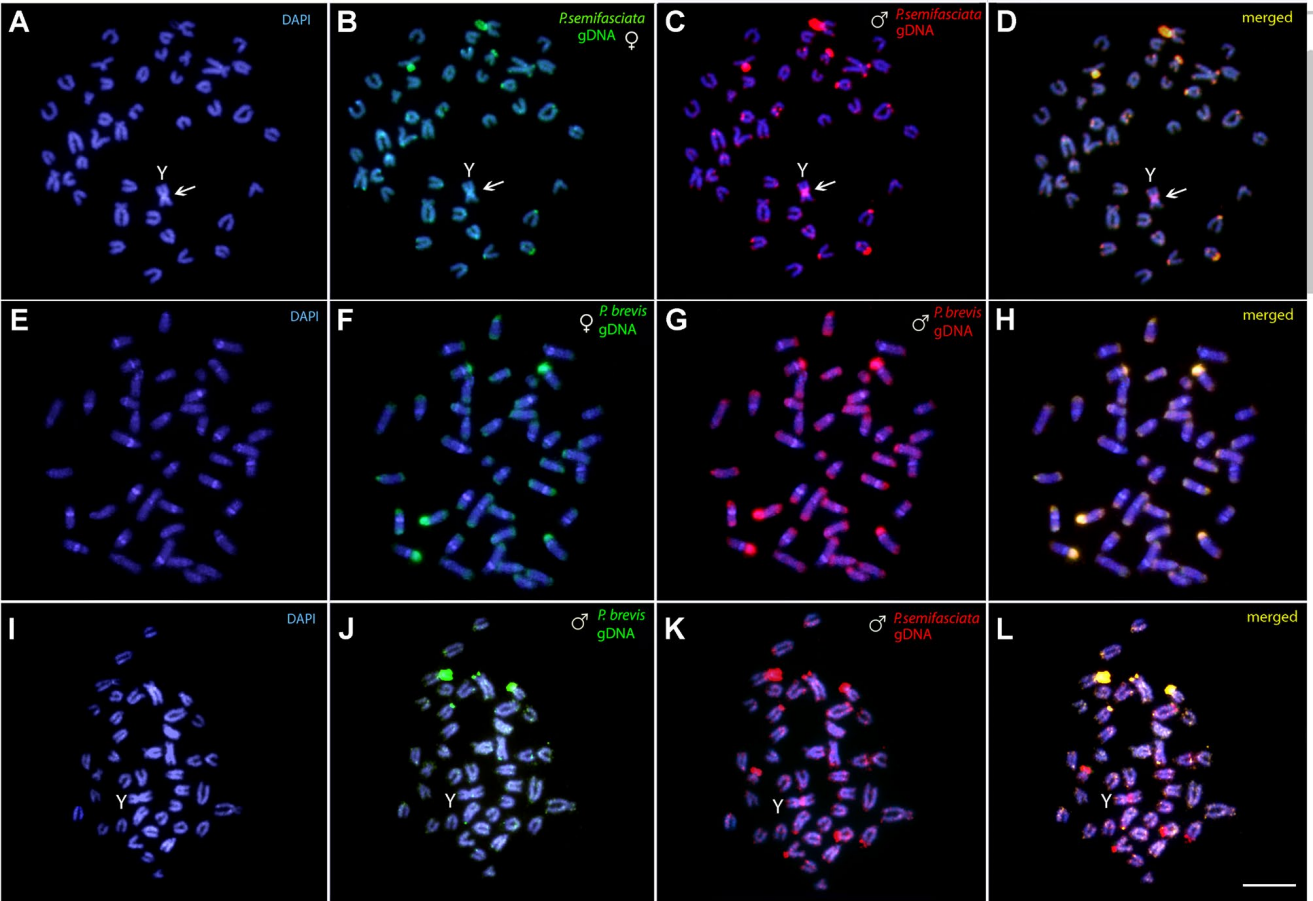
Comparative genomic hybridization (CGH) for intra- and interspecific comparisons. **(A**–**D)** Male- and female-derived genomic probes from *P. semifasciata* mapped against the male chromosomes of *P. semifasciata*. **(E**–**H)** Male- and female-derived genomic probes from *P. brevis* mapped against the male chromosomes of *P. brevis*. **(I**–**L)** Male-derived genomic probes from both *P. semifasciata* and *P. brevis* hybridized together onto male chromosomes of *P. semifasciata*. The common genomic regions of both compared karyomorphs are depicted in yellow and the arrows indicate the male-specific region located on the Y chromosome of *P. semifasciata*. Bar = 5 µm.

The intraspecific genomic hybridization between males and females of *P. semifasciata* revealed a strong binding preference for the male-derived probe to the pericentromeric region of the neo-Y chromosome, while the female-derived probe produced only a weak hybridization signal in this segment (**Figures 6A–D**). The intraspecific genomic hybridization between males and females of *P. brevis* did not show clustering of sex-specific sequences on any chromosome (**Figures 6E–H**).

The interspecific CGH experiments performed to compare the genomes of *P. semifasciata* and *P. brevis* on the level of repetitive DNA divergence yielded a range of non-overlapping species-specific signals as a consequence of their specific evolutionary history. Preferential hybridization of the *P. brevis*-derived probe to the terminal regions of some chromosomes highly likely overlaps with the rDNA sites (**Figures 6I–L**).

### WCP With a PSEMI-Y Probe

**Figure 7:**
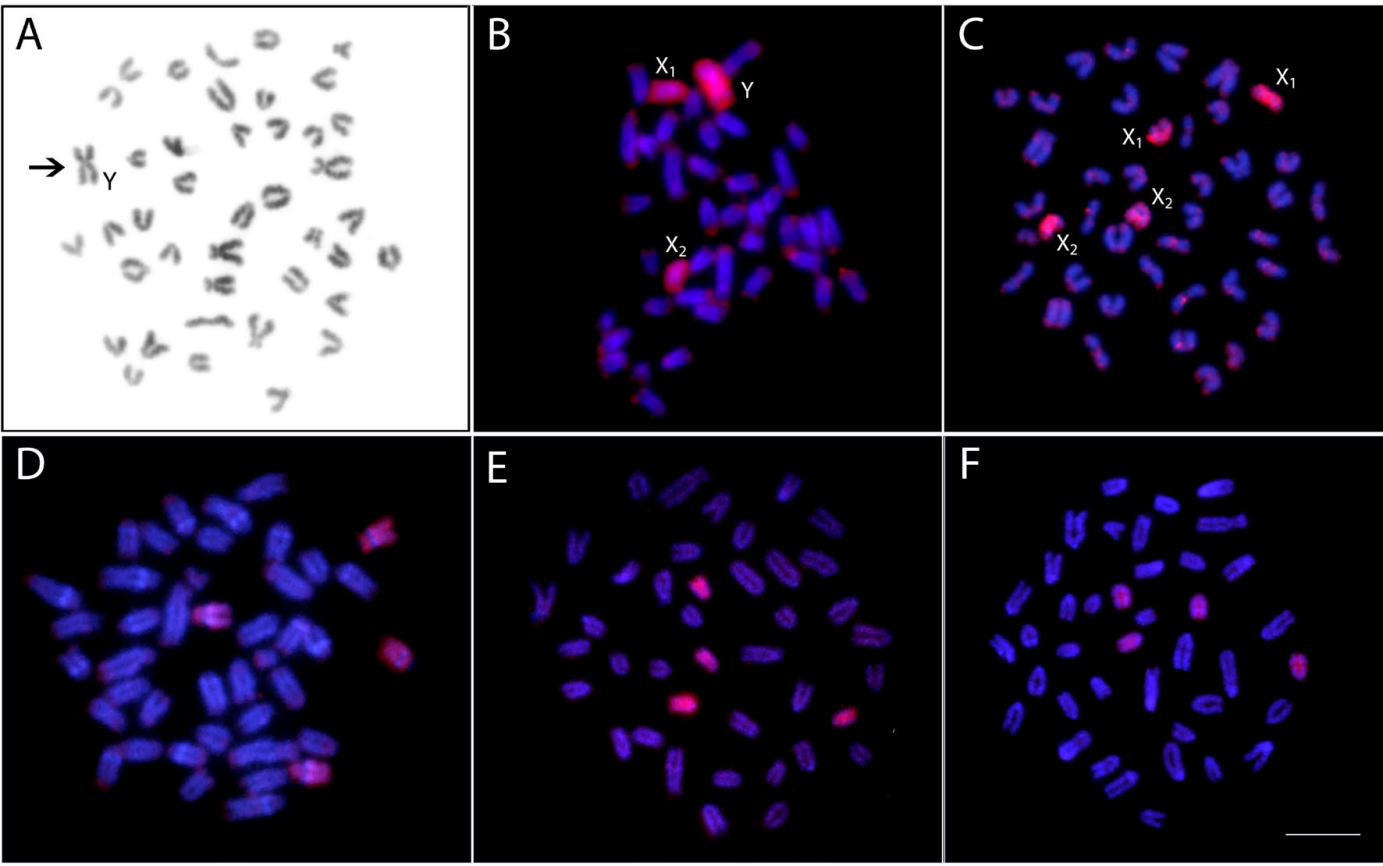
Zoo-FISH with the *PSEMI-Y painting probe* derived from the Y chromosome (arrow) of *P. semifasciata* **(A)** hybridized on the metaphase plates of *P. semifasciata* male **(B)**, *P. semifasciata* female **(C)**, *P. brevis* male **(D)**, *P. australis* male **(E)**, and *Pyrrhulina* aff. *australis* male **(F)**. Bar = 5 µm.

The WCP experiments with the PSEMI-Y probe prepared from the neo-Y chromosome of *P. semifasciata* (**Figure 7A**) entirely painted four chromosomes (named X_1_ and X_2_, two homologs of each) in females and three elements (named X_1_, X_2_, and neo-Y chromosome) in males of* P. semifasciata*, confirming the occurrence of a multiple X_1_X_2_Y sex chromosome system in this species (**Figures 7B, C**). In the cross-species experiments, the PSEMI-Y probe painted two independent chromosome pairs in both males and females of *P. brevis, P. australis,* and* P*. aff. *australis* (**Figures 7D–F**).

## Discussion

### Karyotype and Repetitive DNA Patterns in the Genus *Pyrrhulina*

In many fish groups with taxa of the small-sized body, the lack of cytogenetic data impairs the knowledge about the chromosomal relationships and it prevents to make any meaningful inferences about the impact of chromosome dynamics on their evolutionary history (Liu et al., 2012). The present study brings new insights into the karyotype dynamics of two *Pyrrhulina* species (*P. brevis* and *P. semifasciata*) using conventional and molecular cytogenetic procedures. The karyotype analyses showed the predominance of acrocentric chromosomes in both species, thus documenting a common pattern found in all other studied species from the Lebiasinidae family (Oliveira et al., 1991; Arai, 2011; Moraes et al., 2017). In addition, the observed 2n (41 or 42) fits the conserved 2n found in *Pyrrhulina* species to date, as it ranges from 40 to 42 chromosomes (Oliveira et al., 1991; Arai, 2011; Moraes et al., 2017). Nonetheless, the difference in 2n between male and female in *P. semifasciata* (♂41/♀42) stands out due to the presence of multiple X_1_X_2_Y sex chromosome system, until now unique for this genus. Based solely on the Giemsa-stained karyotypes, an apparent Robertsonian (Rb) translocation gave rise to the largest metacentric Y sex chromosome in the male karyotype. Karyotypes of both analyzed species are otherwise very similar, being composed of 4st + 38a in *P. semifasciata* females and 2sm + 4st + 36a in both sexes of *P. brevis*, as well as in other two *Pyrrhulina* species (*P. australis* and *P.* aff. *australis*), whose karyotypes were revised by us recently, both presenting 4st + 38a (Moraes et al., 2017). This scenario thus points to the involvement of structural chromosome rearrangements such as pericentric inversions in the differentiation of *Pyrrhulina* karyotypes.

Reciprocal interspecific CGH patterns encountered in *P. semifasciata* and *P. brevis* showed that a certain degree of the genome divergence is apparent between both genomes despite close evolutionary relationships between these congeners – on the level of repetitive DNA distribution, manifested by a presence of certain species-specific CGH signals. In addition, such divergent evolutionary features are also supported by the patterns of C-banding and repetitive DNA mapping, in which an advanced stage of sequence divergence is observed, except for the bright signal, corresponding to C-positive heterochromatic/NOR sites (**Figure 6**).

In fact, the presence of interstitial C-bands differentiates *P. brevis* from *P. semifasciata*, in addition to a pool of repetitive elements in such regions that are not shared between these two species, as evidenced by CGH experiments. On the contrary, interstitial C-bands represent a shared trait between *P. brevis* and *P.* aff. *australis* (Moraes et al., 2017) and their presence supports our view about the probable action of intrachromosomal rearrangements of the peri/pericentromeric inversion type in these genomes. Interestingly, a conspicuous polymorphic block of constitutive heterochromatin found previously on the chromosome pair no. 5 in both males and females of *P.* aff. *australis* is not present in the species analyzed herein.

Our hypothesis about the involvement of peri- or paracentric inversions in the karyotype differentiation of *P. semifasciata* and *P. brevis* is further strengthened by the patterns of rDNA distribution. More specifically, the presence of two 5S rDNA sites on the same specific chromosomes in *P. brevis* might indicate that a portion of an original 5S rDNA cluster might have been shifted by inversion to a different location, resulting in a secondary site, similarly to what has been proposed in other (not only) fish groups (Fernandes et al., 2017; Sember et al., 2018a). Nonetheless, bearing in mind that i) the region between doubled 5S rDNA sites encompasses the centromere (thus favorizing pericentric inversions as the underlying mechanism of rDNA mobility) and that, ii) both studied species exhibit very similar karyotypes, an alternative explanation operating with the spreading of 5S rDNA sites through (retro-) transposition is equally probable, especially when taking into account previously reported association of *Rex3* non-LTR retrotransposon with amplified 5S rDNA loci in *P. australis* and *P*. aff. *australis* (Moraes et al., 2017). Taken together, *Pyrrhulina* species deviate from the prevalent patterns of rDNA distribution in fish genomes, where the most of species often bear a single pair of 5S and/or 45S rDNA sites (Gornung, 2013; Sochorová et al., 2018). Nevertheless, multiple rDNA loci are not uncommon in fishes and they might eventually point to elevated genome dynamics, possibly associated with an ongoing interspecific divergence or with the fast fixation due to genetic drift in small populations (Symonová et al., 2013; Sember et al., 2015; Symonová and Howell, 2018). Similar syntenic association of both rDNA classes, as revealed in *P. brevis*, is repeatedly emerging across the teleost phylogeny, being likely rather a by-product of sub-chromosomal dynamics, though bearing potentially some significance with respect to spatial gene co-expression in interphase nuclei, organized into active and inactive domains (Cavalli and Misteli, 2013; Fraser et al., 2015).

Microsatellites are also repetitive elements useful for analyzing the biodiversity and evolutionary processes among fishes (reviewed in Cioffi et al., 2012a). In fact, clustering of microsatellites might help to trace the level of sub-chromosomal dynamics (Basset et al., 2006) and it might also provide important insights into the processes of sex chromosome differentiation (e.g., Kubát et al., 2008; Pokorná et al., 2011; Kejnovský et al., 2013; Poltronieri et al., 2014). In this study, the (CA)_15_ and (GA)_15_ microsatellite motifs showed similar distributional patterns among *P. semifasciata* and *P. brevis* and this holds true also for other species of the *Pyrrhulina* genus already analyzed (Moraes et al., 2017). More specifically, in all four species, the accumulation of (CA)_15_ motif appeared to be almost exclusively telomere-specific, while (GA)_15_ showed rather a dispersed distribution throughout the analyzed chromosome complements, in addition to a higher affinity for telomeric regions. From this data, it might be inferred that microsatellite motifs utilized herein are not resolute for tracking the sub-chromosomal dynamics in *Pyrrhulina,* as their distribution seems to be largely conserved within the analyzed species. Furthermore, they do not show any significant sex chromosome-specific accumulations.

In summary, cytogenetic data accumulated for *Pyrrhulina* species (Moraes et al., 2017, this study) point on largely conserved karyotype macrostructure, yet evidencing extensive dynamics on the sub-chromosomal level, i.e., divergence in the accumulation of certain repetitive DNA classes and highly probable presence of genome-specific repeats. The sub-chromosomal dynamics might be likely facilitated by divergent evolutionary histories of *Pyrrhulina* species and by their common endemic status (Netto-Ferreira and Marinho, 2013).

### Origin and Differentiation of the X_1_X_2_Y Sex Chromosome System in *Pyrrhulina semifasciata*

Although the different 2n present in males (41) and females (42) could also indicate the occurrence of an X0 sex system, our CGH and particularly WCP results confirmed the occurrence of a multiple X_1_X_2_Y sex chromosome system in *P. semifasciata*. Though fishes possess an amazing variety of sex determination and differentiation mechanisms (Devlin and Nagahama, 2002; Herpin and Schartl, 2015; Schartl et al., 2016; Guiguen et al., 2019), sex chromosomes have been described only in about 5% of cytogenetically analyzed species (based on Arai, 2011). It is, however, increasingly apparent that this information is skewed by frequent presence of homomorphic (i.e., cytogenetically unrecognizable) fish gonosomes, which goes hand in hand with their relative evolutionary “youth” and predisposition to frequent sex chromosome turnovers in closely related species or even within species (Kitano and Peichel, 2012; Pennell et al., 2015; Gamble, 2016; Schartl et al., 2016). In spite of that, at least eight sex chromosome systems (XY and ZW and their variations) are known to occur in certain fish species, fully represented also in Neotropical ichthyofauna (Cioffi et al., 2012b; Cioffi et al., 2017). Among them, X_1_X_2_Y gonosomes represent the most frequent multiple sex chromosome system (Kitano and Peichel, 2012; Pennell et al., 2015). In most of the fish taxa with X_1_X_2_Y sex chromosomes, centric or tandem fusions are hypothesized to be the underlying mechanism of their origin, giving rise to a large neo-Y chromosome. Examples of this scenario can be found in several fish species, such as *Harttia punctata* (Loricariidae) (Blanco et al., 2014), *Eigenmannia trilineata* (Sternopygidae) (Fernandes et al., 2010), *Achirus achirus* (Achiridae) (Bitencourt et al., 2016), *Erythrinus erythrinus* and *Hoplias malabaricus* (Erythrinidae) (Bertollo et al., 2004; Cioffi et al., 2013), and *Gymnotus pantanal* (Gymnotidae) (Margarido et al., 2007), among others. Based on available data, it seems likely that chromosome rearrangements are often the fully sufficient mechanism to establish the recombination arrest in different fish neo/multiple sex chromosomes, without need for additional repetitive DNA and heterochromatin accumulation (Almeida-Toledo and Foresti, 2001; Oliveira et al., 2008; Fernandes et al., 2010; Cioffi et al., 2011a; Soares et al., 2014; Cardoso et al., 2015; Sember et al., 2015; Bitencourt et al., 2016; Sember et al., 2018b). This scenario sharply contrasts with several examples in animal or plant kingdom, where massive repetitive DNA accumulations are observed on nascent neo-sex chromosomes (e.g., Mariotti et al., 2009; Bachtrog, 2013).

Bearing in mind its general principle, CGH method might represent a useful tool also for delimitation and gross molecular characterization of sex-specific regions on sex chromosomes and, in many cases, it was also sensitive enough to reveal morphologically homomorphic sex chromosomes (Traut et al., 1999; Symonová et al., 2015; Montiel et al., 2017; Yano et al., 2017b; Freitas et al., 2018; Oliveira et al., 2018; Sember et al., 2018b; Zrzavá et al., 2018). In this study, CGH revealed a notable bias in the accumulation of male-specific or male-enriched repetitive DNA in the pericentromeric region of the Y chromosome, when compared to the intensity of female probe hybridization in the same region. We suppose that this pattern might reflect the incipient stage of differentiation inside the male-specific region, similarly to what has been supposed for karyomorphs C and F of the wolf fish *H. malabaricus* (Freitas et al., 2018; Oliveira et al., 2018; Sember et al., 2018b). It is of interest to note that the region in question encompasses the area around the fusion point on Y. As the recombination in the rearranged region might be significantly reduced or abolished due to sterical constraints, this region gradually accumulates sequence divergence (Faria and Navarro, 2010; Guerrero and Kirkpatrick, 2014). One of the consequences might be a selective advantage, especially if the rearrangement brings into close proximity two (or more) loci whose maintained linkage disequilibrium is favorable to contribute to local adaptation and/or perhaps to speciation (Kawakami et al., 2011) or to resolve genomic conflict (through the linkage of sexually antagonistic genes to male-specific region) (Charlesworth et al., 2005; van Doorn and Kirkpatrick, 2010). The evidence is currently mounting for such scenarios, especially in conjunction with emerging neo-sex chromosomes (Kitano et al., 2009; Yasukochi et al., 2011; Nguyen et al., 2013; Smith et al., 2016; Bracewell et al., 2017). It would be therefore interesting to further investigate, whether the formation of X_1_X_2_Y sex chromosomes was a selected event providing an advantage for a species or whether a genetic drift, highly likely acting in small isolated *P. semifasciata* populations, drove fast fixation of this sex chromosome system randomly just in this species (Charlesworth and Wall, 1999). Importantly, analogous male-*vs*.-female CGH experiments in other *Pyrrhulina* species failed to show any sex-specific region (data not shown).

Lastly, we employed WCP with the PSEMI-Y probe in order to evaluate our hypotheses about the origin of the sex chromosome system present in *P. semifasciata* and to map the orthologous regions in other *Pyrrhulina* species in an attempt to predict potential homomorphic sex chromosomes in these species. Indeed, this approach facilitated many times a finer-scale survey of fish sex chromosomes with a common (Machado et al., 2011; Parise-Maltempi et al., 2013; Pansonato-Alves et al., 2014; Scacchetti et al., 2015; Yano et al., 2017b; Barros et al., 2018) or independent (Reed et al., 1995; Phillips et al., 2001; Henning et al., 2008; Henning et al., 2011; Cioffi et al., 2011b; Cioffi et al., 2013; Oliveira et al., 2018) origin within the frame of certain family, genus, or species/species complex. Here, WCP with the PSEMI-Y probe applied back against its own chromosome complement painted not only the entire metacentric Y chromosome in males but also the entire acrocentric X_1_ and X_2_ chromosomes in both male and female karyotypes. In the cross-species experiments, the PSEMI-Y probe marked four acrocentric chromosomes in both males and females of the other tested *Pyrrhulina* species. These results not only strongly support the proposed origin *via* centric fusion between two non-homologous acrocentric chromosomes, but also that this event might have been fixed in *P. semifasciata* relatively recently, as WCP revealed preservation of all orthologous chromosomes in related *Pyrrhulina* species without apparent major divergence or rearrangements. As CGH results did not show clustering of sex-specific sequences on any chromosome of the *P. brevis* complement, it remains to be investigated, whether any of four PSEMI-Y labeled chromosomes represent cryptic (homomorphic) sex chromosomes with a sex-specific region being under resolution limit of the CGH method, or whether sex chromosomes are not present at all in this species and the sex determination is governed by other means (Herpin and Schartl, 2015; Guiguen et al., 2019).

## Conclusion

Despite methodological difficulties, sufficient chromosomal preparations were obtained in miniature fishes of the genus *Pyrrhulina* in the present study. It was possible to demonstrate that chromosomal markers are useful cytotaxonomic tools in characterizing the biodiversity of these fishes, highlighting their evolutionary relationships. Among the obtained results, a discovery of multiple sex chromosome system in our *P. semifasciata* stands out and its investigation have led us to the following conclusions: i) the neo-Y chromosome arose likely from a centric fusion between two non-homologous acrocentric chromosomes or, possibly, between former (proto) sex chromosomes and an autosomal pair; ii) this event might have been fixed in *P. semifasciata* relatively recently, as revealed by CGH and WCP; the latter technique revealed all orthologous chromosomes in related *Pyrrhulina* species without apparent major divergence or rearrangements; iii) formation of neo-Y in *P. semifasciata* might be driven by genetic drift, while direct selective/adaptive advantage resulting from close association of formerly unlinked genetic content cannot be ruled out; and iv) despite presumably short evolutionary time, CGH revealed considerable accumulation of male-enriched sequences in the pericentromeric region of neo-Y. Whether the origin of multiple sex chromosomes was driven by positive selection or by genetic drift and whether related cryptic sex chromosomes occur in sibling species, remains to be shown. Nonetheless, a nascent male-specific region on Y in *P. semifasciata* as might be inferred from CGH suggests fast sequence evolution, with the area around the fusion point potentially hosting candidate genes for the sex determination.

The present study further underscores the importance of analyzing data from so-called lower vertebrates such as fishes, as the evolutionary scenarios uncovered in these lineages may provide important clues about the fundamental processes behind the genome organization and the karyotype evolution of vertebrates in general. It may particularly increase our cytogenetic knowledge in so-called higher vertebrates, especially when we take into account that intra- and interchromosomal rearrangements are potent drivers of evolution in Hominoidea, with gibbons of the family Hylobatidae representing the most spectacular example (Weise et al., 2015, Sangpakdee et al., 2016). The same holds true for the dynamics of repetitive DNA, which significantly contributes to karyotype divergence among fishes, but is rarely studied in detail in higher vertebrates (Mrasek et al., 2001; Liehr et al., 2016), despite it might play a relevant role in species’ divergence here as well. Finally, also complex sex-chromosome systems, such as the one described in the present study, hold a great potential to build up the reproductive barriers among different populations of the same species and can be occasionally found also in Hominoidea, as exemplified, e.g., by *Trachypithecus cristatus* (Xiaobo et al., 2013).

## Data Availability

All datasets generated for this study are included in the manuscript.

## Ethics Statement

The experiments followed ethical and anesthesia conducts and were approved by the Ethics Committee on Animal Experimentation of the Universidade Federal de São Carlos (Process number CEUA 1853260315).

## Author Contributions

RM and AS carried out the cytogenetic analysis and drafted the manuscript. TH, EO, AA-R, and PV helped in the cytogenetic analysis, drafted and revised the manuscript. TL, PR, EF and MM drafted and revised the manuscript. MC and LB coordinated the study, drafted and revised the manuscript. All authors read and approved the final version of the manuscript.

## Funding

This study was supported by Conselho Nacional de Desenvolvimento Científico e Tecnológico – CNPq (Proc. nos 401575/2016-0 and 302449/2018-3), Fundação de Amparo à Pesquisa do Estado de São Paulo- FAPESP (Proc. Nos. 2015/26322-0 and 2017/09321-5) and CAPES/Alexander von Humboldt (Proc. No. 88881.136128/2017-01). Further, by the project EXCELLENCE CZ.02.1.01/0.0/0.0/15_003/0000460 OP RDE and with the institutional support RVO: 67985904 (AS, PR) and PPLZ: L200451751 (AS).

## Conflict of Interest Statement

The authors declare that the research was conducted in the absence of any commercial or financial relationships that could be construed as a potential conflict of interest.

The handling editor declared a past co-authorship with one of the authors TL.
